# Optic coherence tomography features of subretinal vitreous substitutes

**DOI:** 10.1186/s40942-020-00256-7

**Published:** 2020-11-13

**Authors:** Leandro Cabral Zacharias, Epitácio Dias da Silva Neto, Taurino dos Santos Rodrigues Neto, José Ronaldo Lima de Carvalho Júnior, Rony Carlos Preti, Leonardo Provetti Cunha, Tatiana Tanaka, Mário Luiz Ribeiro Monteiro

**Affiliations:** Division of Ophthalmology, Medical School, University of São Paulo, Rua, Av. Dr. Enéas Carvalho de Aguiar, 255, Cerqueira César, São Paulo, SP 05403-000 Brazil

**Keywords:** Gas tamponade, Ophthalmologic surgical procedures/methods, Perfluorocarbon liquid, Retinal detachment/surgery, Silicone oils/therapeutic use, Vitrectomy

## Abstract

**Background:**

To draw comparisons between spectral domain optic coherence tomography (SD-OCT) features of subretinal silicon oil (SO), perfluoro-*n*-octane (PFO) or C3F8 gas.

**Methods:**

Cases diagnosed with retained subretinal vitreous substitutes (VS) were retrospectively selected. Demographic data were collected and OCT features were analyzed.

**Results:**

In the 13 cases with subretinal PFO, hyper-reflectivity under the bubble was noted in 8 eyes (61.5%); choroidal shadow at the borders of the bubble in 11 eyes (84.6%); hyper-reflective halo around the bubble in 5 eyes (38.4%) and a hyper-reflective apical dot in 8 eyes (61.5%).The two cases with multiple PFO bubbles had complete septum dividing the bubbles. The one case with subretinal SO had hyper reflectivity under the bubble; no choroidal shadow at the edge of the bubble; hyper-reflective halo was noted around the bubble and the apical hyper-reflective dot was present; there was no complete septum dividing multiple bubbles. The single case with subretinal C_3_F_8_ had some bubbles with totally round base, incomplete septum, hyper reflectivity under the bubble, choroidal shadow at the edge of the bubble, a hyper-reflective halo and an apical dot.

**Conclusion:**

Different subretinal VS share similar SD-OCT characteristics. Round base bubbles are only observed with subretinal C3F8 gas, while incomplete septum are related to retained subretinal SO or gas.

## Background

Perfluoro-n-octane liquid (PFO) is widely used in vitreoretinal surgery for retinal detachment (RD) repair due to its specific characteristics, that include high density, low surface tension, low viscosity and optical clarity [1]. One possible complication of PFO use is its migration to the subretinal space, which may lead to retinal structural and functional changes [2–4]. That complication may occur in up to 2% of cases even when PFO is carefully manipulated [5]. When the PFO bubble involves the fovea, surgical removal should be considered.

Other vitreous substitutes (VS), such as silicone oil (SO) or gas, have been used for decades as tamponades after RD repair. Multiple reports have described spectral-domain optical coherence tomography (SD-OCT) features of emulsified subretinal SO [6, 7], but SD-OCT features of non-emulsified subretinal SO, to the best of our knowledge, are yet to be described. Subretinal gas migration has already been described [8], but not its SD-OCT features.

SD-OCT aspects of retained subretinal PFO have been described and help distinguish PFO from residual subretinal fluid, macular cysts or cystoid macular edema [9, 10], but no attempts have been made in literature to distinguish between subretinal retained PFO, SO or gas.

This paper was motivated by two patients that were erroneously interpreted as having subfoveal PFO, but in fact had subretinal SO or gas verified intraoperatively. We therefore performed a retrospective analysis of the SD-OCT features of patients with retained subretinal vitreous substitutes (VS). The purpose of this paper is to describe the SD-OCT features of retained subretinal VS and to draw comparisons between SD-OCT features of subretinal SO, PFO or gas.

## Methods

Cases diagnosed with retained subretinal VS were retrospectively selected based on SD-OCT images. All selected patients had the diagnosis of rhegmatogenous RD and were operated on between August 2006 and July 2016 at the department of Ophthalmology- the University of Sao Paulo medical school.

Patients were excluded from the analysis if no high quality SD-OCT image was obtained or if there was no certainty on which subretinal VS was imaged. The SD-OCT examination was performed using the Heidelberg Tracking Laser System (Heidelberg Engineering, Heidelberg, Germany). Demographic data were collected and the following features on OCT imaging were analyzed: hyper-reflective elevation of the subretinal vitreous substitute/RPE interface; hyper-reflectivity of the choroid under the VS bubble; shadow in the choroid at the borders of the VS bubble; hyper-reflective retinal halo around the bubble; bubble shape (totally round vs flat base); and hyper-reflective apical dot. In cases of multiple bubbles, the presence of septum dividing the bubbles was also analyzed. We considered that a complete septum was present if it touched the RPE in any scan analyzed.

Data were collected from 15 eyes of 15 patients. Patient’s median age was 49.35 years old (range 13–76). Seven right eyes (46.7%) and 8 (53.3%) left eyes were included. All patients were submitted to 23-gauge vitrectomy for rhegmatogenous retinal detachment repair. Thirteen patients had retained subretinal PFO, one patient had retained SO and one had retained subretinal C_3_F_8_.

## Results

Data were collected from 17 eyes of 17 patients. Two patients were excluded from the analysis due to poor OCT imaging. The postoperative OCT scans from 15 patients (9 male and 6 female) were analyzed. Patient’s median age was 49.35 years old (range 13–76). Seven right eyes (46.7%) and 8 (53.3%) left eyes were included. All patients were submitted to 23-gauge vitrectomy for rhegmatogenous retinal detachment repair. Thirteen patients had retained subretinal PFO, one patient had retained SO and one had retained subretinal C_3_F_8_.

OCT analysis demonstrated that the elevation of the vitreous substitute/RPE interface and flat shape of the bubble was noted in all cases despite of which vitreous substitute was present. In the 13 cases with subretinal PFO, the hyper-reflectivity under the bubble was noted in 8 eyes (61.5%); shadow in the choroid at the borders of the VS bubble was noted in 11 eyes (84.6%); a hyper-reflective halo around the VS bubble was present in 5 eyes (38.4%) and a hyper-reflective apical dot in 8 eyes (61.5%). The two cases with subretinal PFO and multiple bubbles had complete septum dividing the bubbles. The one case with subretinal silicon oil had no hyper-reflectivity under the bubble and no choroidal shadow at the edge of the bubble; hyper-reflective halo was noted around the bubble and an apical hyper-reflective dot was present; there was no complete septum dividing the multiple bubbles. The single case with sub retinal C_3_F_8_ had hyper-reflectivity under the bubble and choroidal shadow at the edge of the bubble; the hyper-reflective halo and the apical dot were both present. Only in this particular case, some totally round shape bubbles and flat base bubbles were both noted; there was no complete septum dividing the multiple bubbles (Table 1).

**Table 1:** Spectral-domain optical coherence tomography (SD-OCT) characteristics of retained subretinal vitreous substitutes

	Perfluoro-*n*-octane	Silicon oil	Gas (C_3_F_8_)
Hyperreflective elevated line between vitreous substitute/RPE	13/13 (100%)	1/1 (100%)	1/1 (100%)
Hyperreflectivity under the bubble	8/13 (61.5%)	1/1 (100%)	1/1 (100%)
Choroidal shadow	11/13 (84.6%)	1/1 (100%)	1/1 (100%)
Hyperreflective halo	5/13 (38.4%)	1/1 (100%)	1/1 (100%)
Bubble shape	Flat base	Flat base	Flat base/round
Hyperreflective apical dot	8/13 (61.5%)	1/1 (100%)	1/1 (100%)
Complete septum (if multiple bubbles)	2/2 (100%)	0/1 (0%)	0/1 (0%)

Three elucidative cases are described below.

### Case 1

**Fig. 1:**
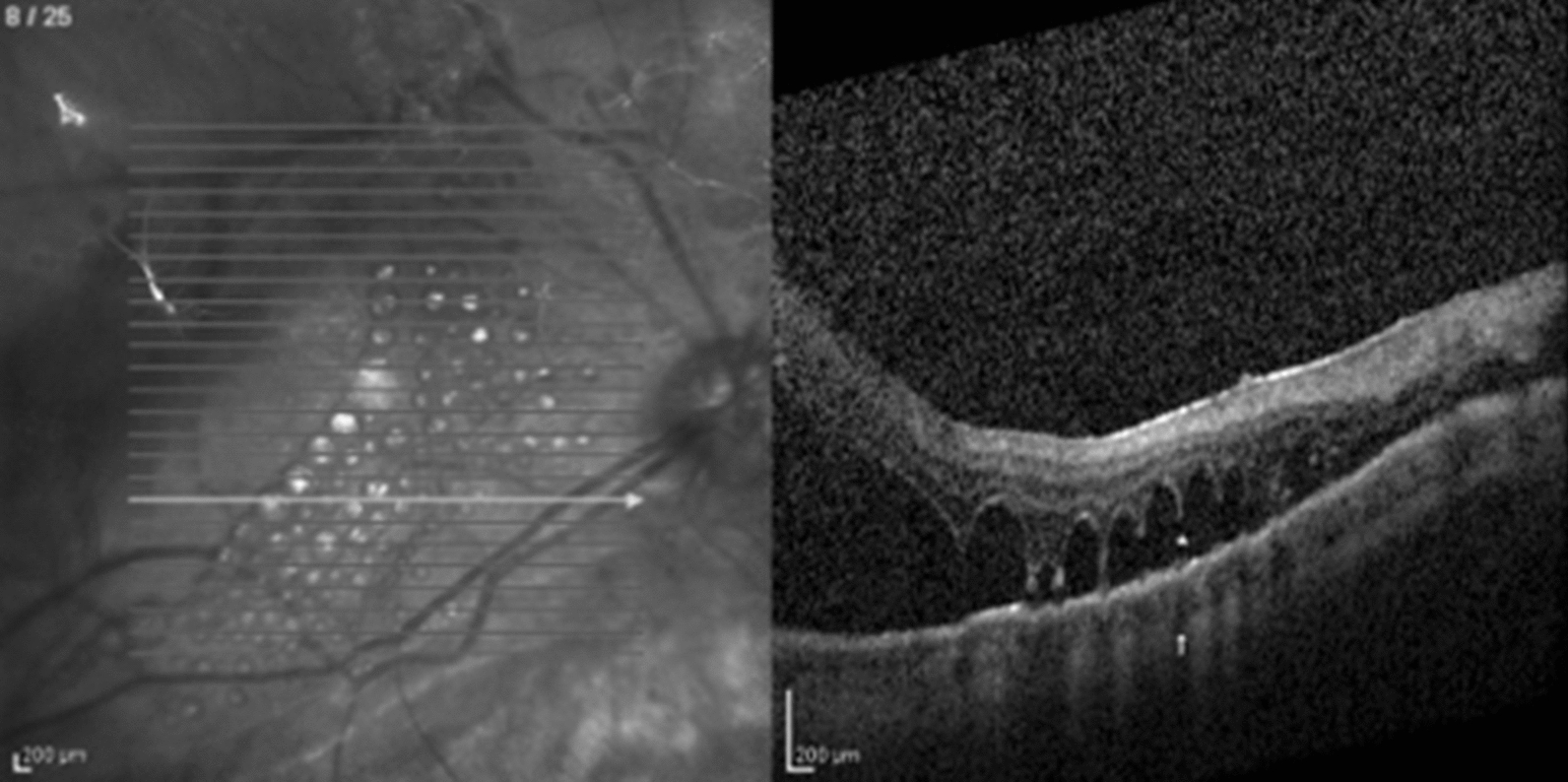
SD-OCT demonstrates subretinal silicone oil. There is no complete septum dividing the multiple bubbles—we named this finding as the “caterpillar sign” (star). Note the choroidal shadow at the borders of the bubbles (white arrow)

A 58 year-old male presented to our service after three vitreoretinal surgeries for recurrent rhegmatogenous retinal detachment and proliferative vitreoretinopathy (PVR) in his right eye. His visual acuity at presentation was count fingers. On ophthalmic examination, the patient was pseudophakic; fundus examination revealed inferior and temporal retinal detachment under silicone oil tamponade, massive PVR, and multiple small sub retinal VS bubbles at the macular region, that were initially considered to be PFO (Fig. 1). Surgery was indicated to flatten the retina, remove pre-retinal membranes and the multiple presumed subretinal PFO bubbles. During the PPV, all pre-retinal membranes were removed. PFO introduced to flatten the retina dislodged the subretinal VS bubbles to the temporal periphery. A peripheral retinotomy was therefore created to drain the subretinal fluid. At this moment, the subretinal VS floated in the vitreous cavity and only then the diagnosis of subretinal SO was made. At the end of the case, retina was totally reattached, laser photocoagulation was performed in all breaks and silicone oil was used as a tamponade. After three months, best-corrected visual acuity improved to 20/200.

### Case 2

**Fig. 2:**
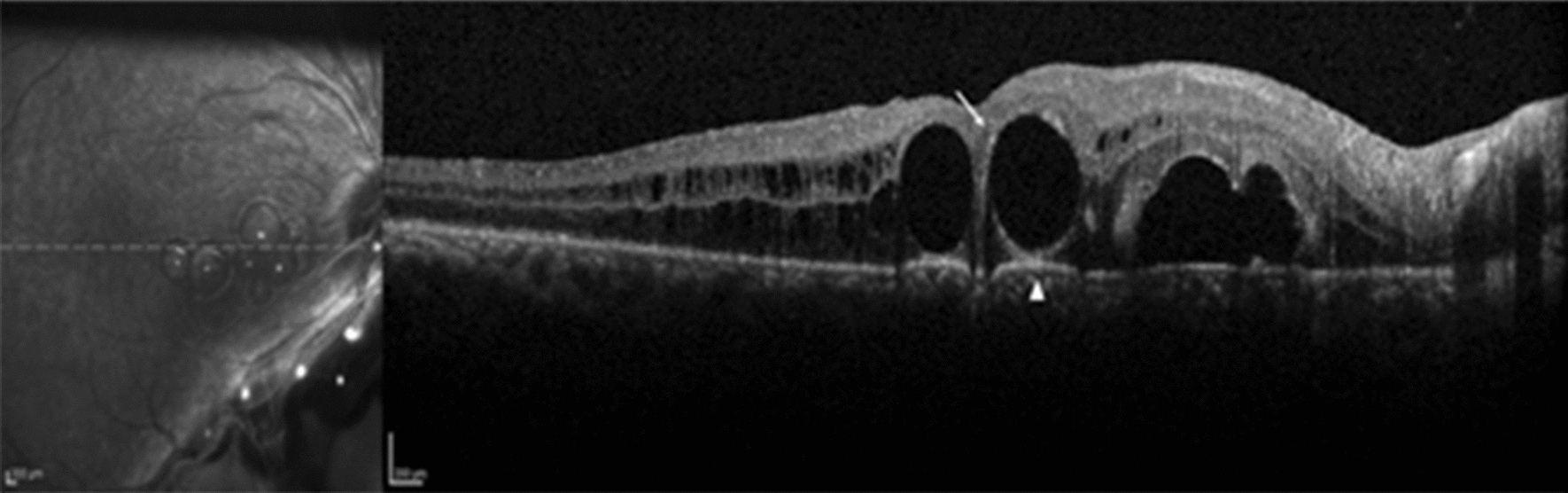
SD-OCT demonstrates subretinal C_3_F_8_ in a case of retinal detachment and optic nerve coloboma. Note hyper-reflective elevated band at the VS/RPE interface and a round base of the bubble above it (arrowhead). Hyper-reflective halo around the retained subretinal gas is also present (arrow)

A 24 year-old male was referred to our service due to sudden visual loss in his left eye over the last 7 days. Past ocular history was positive for amblyopia in his left eye. At presentation, his visual acuity was hand motion; biomicroscopy revealed lens coloboma. Fundus examination revealed total retinal detachment, a superotemporal horseshoe tear, and inferonasal retinochoroidal coloboma. A 23-gauge pars plana vitrectomy was then performed. PFO was used to flatten the retina and 10% C_3_F_8_ was used as a tamponade. After 2 weeks, retina was attached under partially absorbed gas and subretinal macular bubbles were observed (Fig. 2). An erroneous presumption of subretinal PFO was made and the patient was submitted to a new procedure in order to remove the PFO. During surgery, the removal of the subretinal VS bubbles was attempted with a 41-gauge cannula. Minimal manipulation led to the prompt mobilization of the subretinal VS to the retrolental space, and therefore the diagnosis of subretinal gas was achieved. 6 weeks after the second intervention, a recurrent retinal detachment related to PVR was observed. A new vitreoretinal surgery was indicated, but the patient refused any further intervention.

### Case 3

**Fig. 3:**
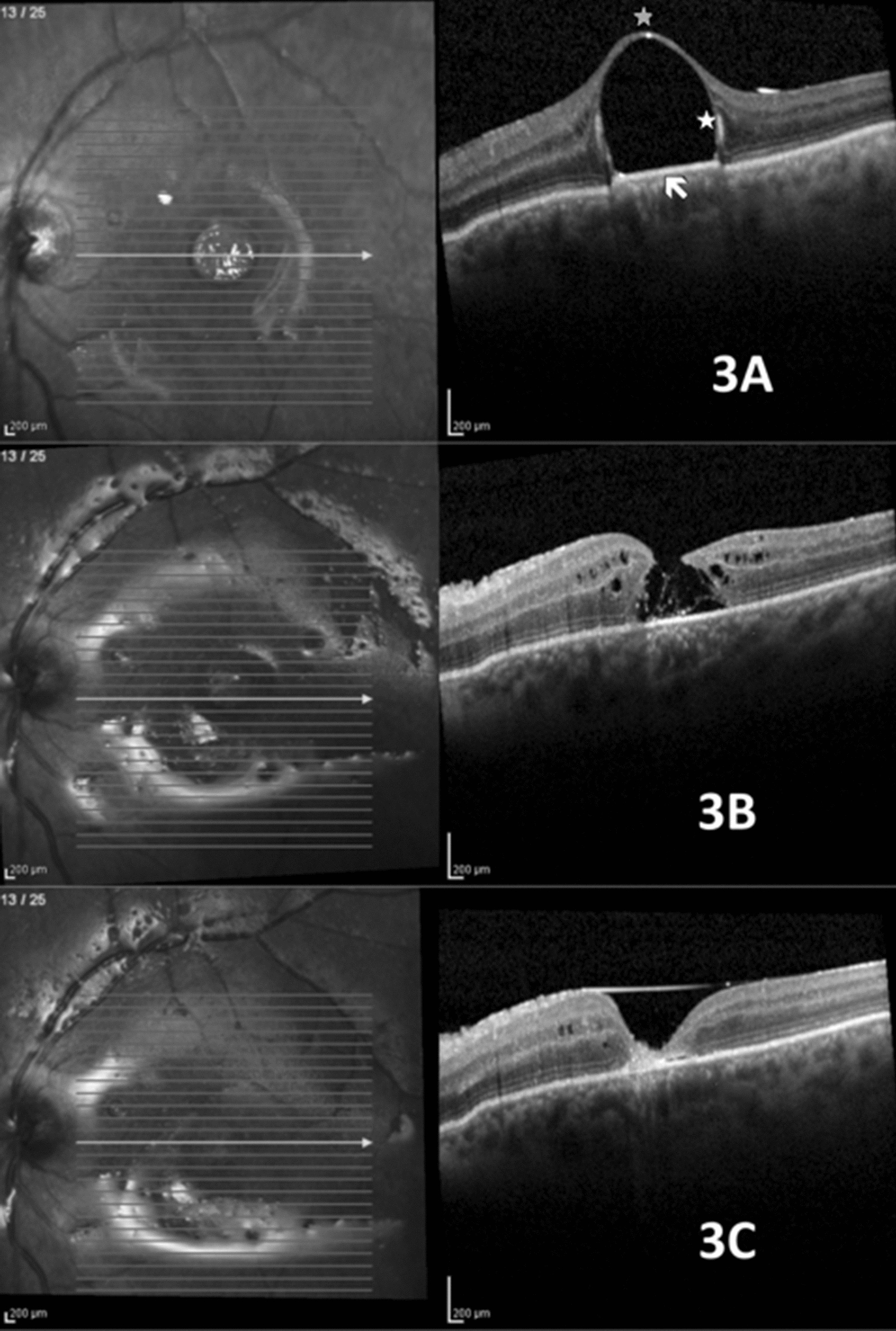
Subretinal PFO under the fovea. **a** Features before surgical removal. Gray asterisk: hyper-reflective apical dot; white asterisk: hyper-reflective halo; white arrow: elevation of PFO/RPE interface. **b** Post op day 1. The RPE elevation is not noted; image resembles full thickness macular hole, but the roof of the cyst connects the borders. **c** Seven days post op. Closure of internal retinal layers; absence of subfoveal external layers (white arrow)

A 61-year old woman was referred to our service one week after a second vitreoretinal surgery for recurrent retinal detachment in her left eye. Ophthalmic examination revealed a BCVA of count fingers, retina attached under silicone oil and a subfoveal PFO bubble (Fig. 3a). Surgery was scheduled in order to remove the subretinal PFO. Considering the short interval between surgeries, a two-port vitrectomy without infusion placement or silicone oil removal was indicated and performed. At post op day one, SD-OCT image revealed discontinuity of the neurosensory retina. A more careful analysis showed a thin collapsed membrane, which was considered to be the roof of the retinal cyst that was previously filled with PFO (Fig. 3b). At post op day 7, OCT showed anatomical improvement (Fig. 3c). After 2 months, best-corrected visual acuity improved to 20/100.

## Discussion

PFO is widely used in vitreoretinal surgeries for complex retinal detachment treatment. Its chemical properties, high specific gravity, optical clarity, low surface tension and low viscosity make it a useful tool for better retinal placement and stabilization in challenging cases such as giant retinal tears, penetrating ocular trauma, or large retinectomies. Although the use of PFO has promoted great advances in the retinal detachment repair, especially for complex cases, the subretinal retention of PFO, especially in the macular area, is one of the most unwanted complications. This complication could happens in approximately 1% of the cases and induces local photoreceptors damage and disruption of external limiting membrane, resulting in localized absolute scotomas. Therefore, prompt surgical removal is indicated when it is located at or near the foveal region [11].

Several maneuvers have already been described to remove subretinal PFO, such as direct transretinal aspiration through a 41-gauge cannula, subretinal fluid infusion causing retinal redetachment and PFO dislodgment; or large retinotomies for subretinal access and direct bubble aspiration [12, 13]. In cases of other subretinal VS that not PFO, observation may be elected when dealing with subretinal gas or a large relaxing retinotomy may be necessary to access the high viscosity silicone oil, that tends to float after released from the subretinal space. Therefore, depending on the type of subretinal VS, different surgical approaches are applied and the previous knowledge of which VS is retained may facilitate surgical planning.

Some SD-OCT features of subretinal PFO have already been described and help distinguish it from cysts or subretinal fluid [9, 10, 14], but no attempt in literature so far tried to differentiate subretinal PFO from gas or SO. Previous attempts to describe OCT features of subretinal SO dealt with emulsified particles, described on OCT as subretinal hyper-reflective spherical bodies, usually at the border of a macular hole [6, 7], but there is no previous literature so far describing SD-OCT features of retained subretinal non-emulsified SO.

Most of the SD-OCT features observed in the present study are similar among different retained subretinal VS, but subtle differences combined with clinical suspicion may help differentiate among them. A hyper-reflective band at the subretinal VS-RPE junction has already been described in cases of subretinal PFO [10, 14]. This sign may be explained by two possible theories: RPE loss due to PFO toxicity; or an optical phenomenon at the interface between two media with different refractive indices (PFO and choroid) [14]. Considering that this sign was present in all three different VS studied, and that it disappears the day after PFO is removed (as shown in Fig. 3), we believe this is an optical phenomenon and the presence of this sign does not help differentiate among the three VS.

**Fig. 4:**
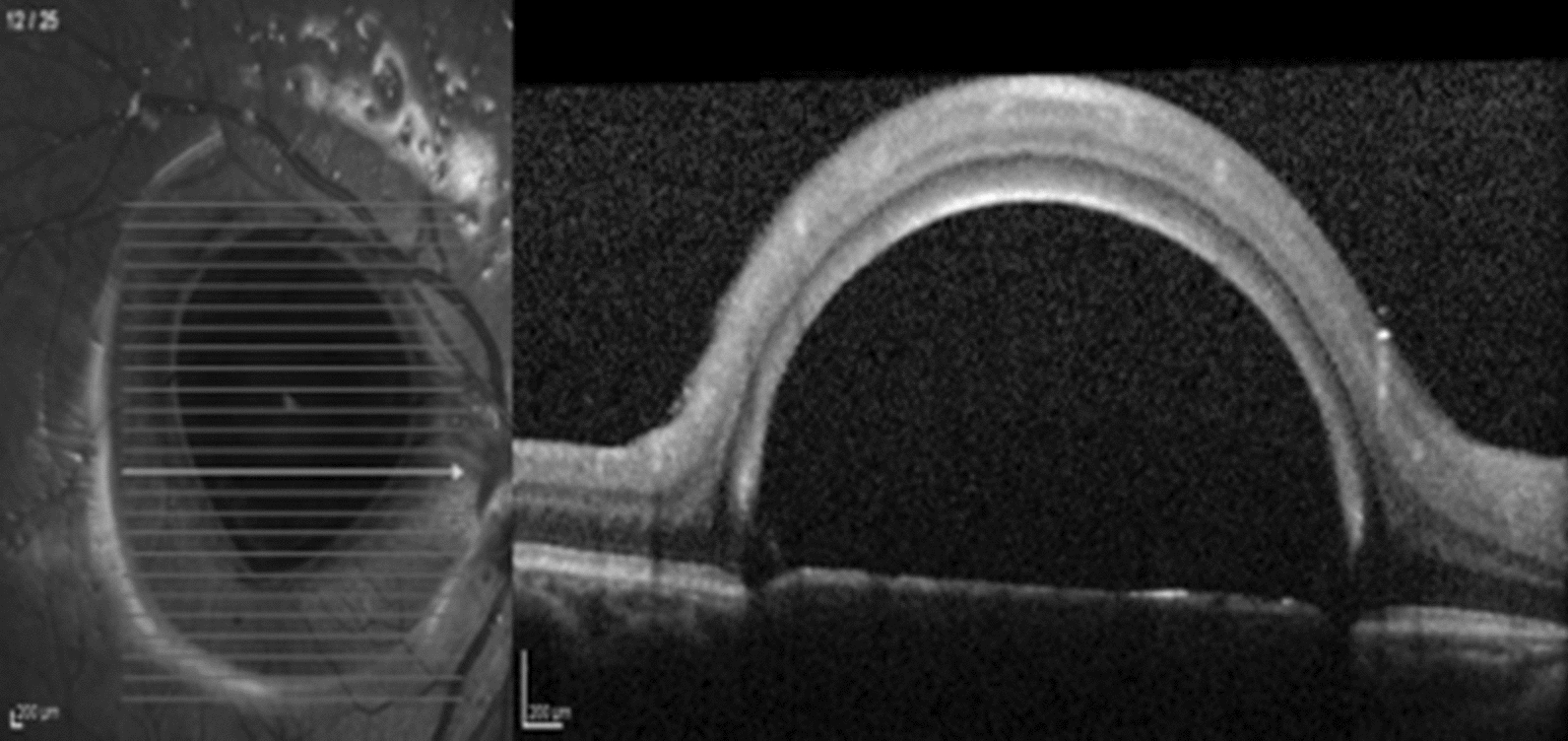
Giant subfoveal PFO bubble. Note the hyper-reflective halo around the flat base bubble, as well as the elevation of the PFO/RPE interface

The pinpoint focus of hyper-reflectance at the apex of the PFO bubble has also been previously described and helps to distinguish PFO from cysts [9]. We have found this sign in most of the retained PFO cases, as well as in cases of gas or SO, and therefore it does not seem to help differentiate among different subretinal VS. A hyper-reflective halo around the bubble at the subretinal space, as described in Figs. 2 and 4, was observed in a small proportion of subretinal PFO cases, but it also does not help to differentiate among other VS, as it was also present in gas and retained subretinal SO bubbles in our series. Conversely, hyper-reflectivity of the choroid under the bubble area and choroidal shadow at the border of the bubbles were observed in most subretinal VS cases and do not help distinguish among different VS.

In some of our cases, the subretinal PFO produces a hyper-reflective shadow obscuring the choroid (Fig. 3a). We have no clear explanation for the presence of this hyper-reflective shadow and why it appears only in some cases, but other authors assume it may be caused by an optical effect [10]. We also carefully analyzed the bubble shape, as we considered that the physical properties of different VS could lead to different outcomes. Multiple scans were perused in order to determine the bubble shape, and all but some subretinal gas bubble showed a flat base. A round base of the bubble seen on SD-OCT seems to be indicative of subretinal gas, especially in the presence of optic disc anomalies, as described previously [8]. Recent studies suggest the use of subretinal injection of tissue plasminogen activator (tPA) and air in massive subretinal hemorrhage [15]. Thus, post-operative images of subretinal air can be more frequently found and better characterized in the OCT exam.

**Fig. 5:**
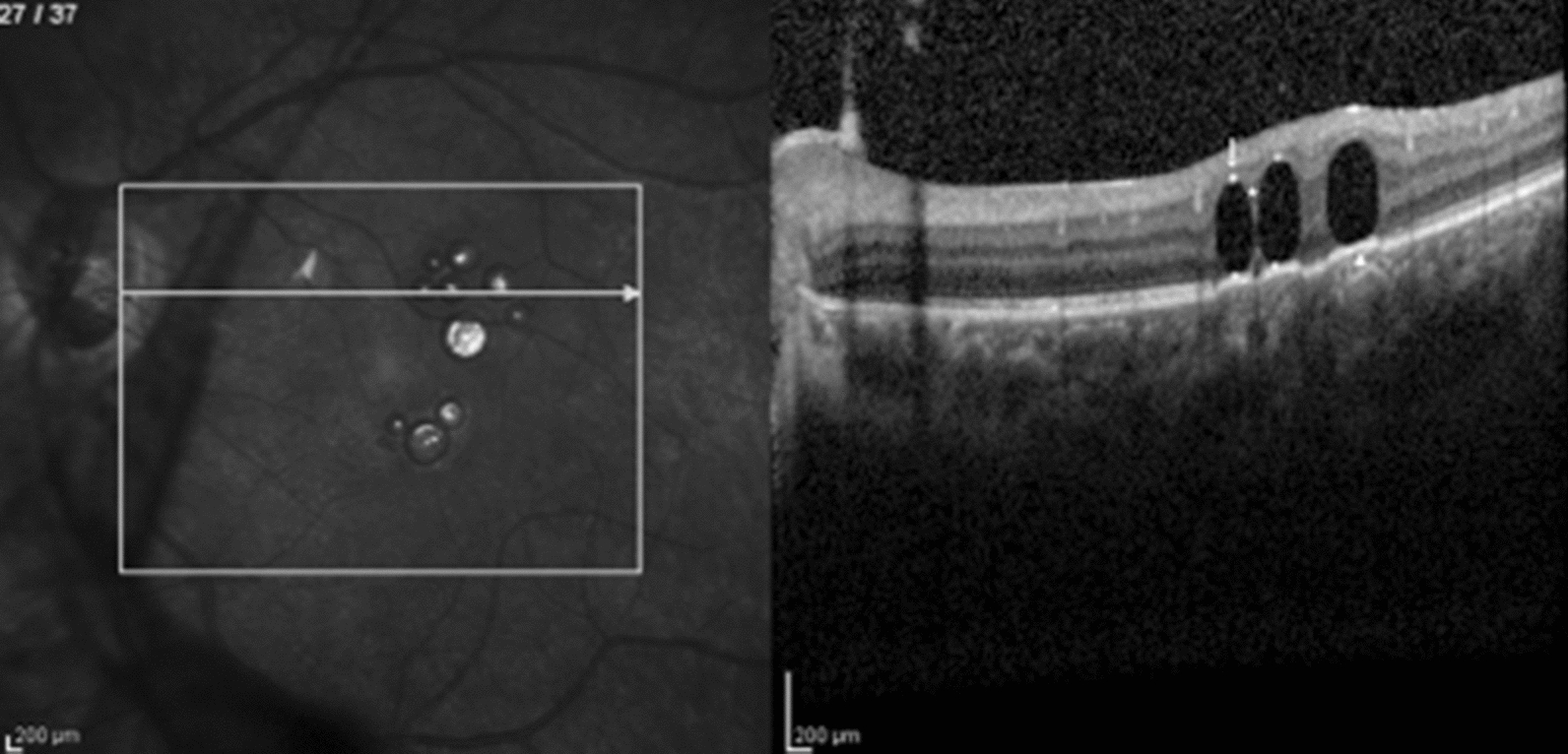
SD-OCT demonstrates multiple retained subretinal PFO bubbles. Note the choroidal hyper-reflectivity under the bubble area and hyper-reflective elevated band at the VS/RPE interface under a flat bubble shape (arrowhead). A hyper-reflective apical dot (arrow) and a septum between the bubbles (star) are also present

Both cases of subretinal PFO with multiple bubbles had complete septum dividing the bubbles, as shown in Fig. 5. This finding was previously described [11], and may be related to the low surface tension and viscosity presented by the PFO, that tend to coalesce into a single bubble when there is no tissue separating it. On the other hand, subretinal SO or gas tend to form multiple bubbles with incomplete septum among them- a SD-OCT feature that we named the “caterpillar sign” (Figs. 1, 2) and may be a helpful hint to differentiate among retained subretinal VS.

The limitation of our study is the small amount of cases in some of the analyzed groups. In our series, there is only one case of each subretinal SO and gas, which limits the generalization of our findings. However, considering that no SD-OCT features of retained subretinal gas or SO (excluding emulsification) have been previously reported and the extremely rare frequency of such occurrences, we believe our observation may be helpful for selected complicated cases. Therefore, further studies with an increased number of patients with retained subretinal SO and gas are needed in other to confirm the findings of our series.

## Conclusion

In cases of subretinal VS retention, it is clinically important to differentiate between PFO, SO or gas. Despite of the small number of cases in the SO and gas groups, this study suggests that different subretinal VS have similar SD-OCT characteristics. In summary, a round base of the bubble was observed with subretinal C3F8, while incomplete septum is related to retained subretinal SO or gas.
